# CyclinD1 Is a New Target Gene of Tumor Suppressor MiR-520e in Breast Cancer

**DOI:** 10.1515/med-2019-0108

**Published:** 2019-12-04

**Authors:** Quan Liang, Qingjuan Yao, GuoYing Hu

**Affiliations:** 1 Department of General Surgery, General Hospital of Tianjin Medical University, Tianjin 300051, China; 2 Central Laboratory, The 2nd Hospital of Tianjin Medical University, Tianjin 300211, China; 3 Tianjin Institute of Urology, Tianjin 300211, China

**Keywords:** CyclinD1, miR-520e, Breast cancer

## Abstract

**Objective:**

To investigate the involvement of miR-520e in the modulation of cancer-promoting cyclinD1 in breast cancer.

**Methods:**

A reverse transcription-polymerase chain reaction (RT-PCR) was applied to test the regulation of miR-520e on cyclinD1. The binding of miR-520e to 3’-untranslated region (3’UTR) of cyclinD1 mRNA was predicted by an online bioinformatics website. The effect of miR-520e on the luciferase reporters with binding sites of miR-520e and 3’UTR of cyclinD1 mRNA was revealed using a luciferase reporter gene assay. The correlation between miR-520e and cyclinD1 in clinical breast cancer samples was detected through quantitative real-time PCR.

**Results:**

The expression of cyclinD1 was gradually reduced as the dose of miR-520e increased. Anti-miR-520e obviously induced cyclinD1 in breast cancer cells. After anti-miR-520e was introduced into the cells, the inhibition of cyclinD1 expression mediated by miR-520e was reversed. The binding of miR-520e with cyclinD1 was revealed via bioinformatics. Under the treatment of dose-increasing miR-520e or anti-miR-520e, the luciferase activities of cyclinD1 3’UTR vector were lower or higher by degrees. However, the activity of the mutant vector was not affected at all. Finally, in clinical breast cancer tissues the negative correlation of miR-520e with cyclinD1 was revealed.

**Conclusion:**

In conclusion, cyclinD1 is a new target of miR-520e in breast cancer.

## 1 Introduction

MicroRNAs can regulate the expression of genes through making mRNA degradation or inhibit translation. During the development of cancer, microRNAs can function as cancer promoters or cancer suppressors. A report has revealed that miR-520e makes breast cancer cells sensitive to complement attacks, resulting in the suppression of breast cancer [1]. Some microarray data shows that lots of microRNAs including miR-520e might be related to the occurrence and development of breast cancer [2]. MiR-520e can have a great part in the modulation of progression of breast cancer [3]. In other studies, miR-520e is found to inhibit hepatocarcinogenesis and interact with NIK in liver cancer [4, 5]. MiR-520e-targeting EphA2 involves the MAPK pathway and then functions in HBV replication or liver cancer growth [6]. Some researchers have made progress in delivering miR-520e for lung cancer therapy [7]. Some analyses of microarray and microRNA profiling reveal an association of miR-520e with JAK1 in lung cancer[8]. MiR-520e can repress cell proliferation, migration and invasion through interacting with Zbtb7a in non-small cell lung cancer [9]. In response to TGF-β, SMAD-activated miR-520e is able to target TGFBR2, resulting in the suppression of NSCLC metastasis [10]. MiR-520e with some other microRNAs is involved in the development of lynch syndrome [11]. In glioma, miR-520e can target FGF19 to inhibit the Wnt/β-catenin pathway for destroying cell growth and invasion [12]. However, the role of miR-520e and its new target still need investigating.

Serving as an important cell cycle driver, the cyclinD1 protein is encoded by the CCND1 gene and it is a prognostic and predictive factor in cancers. Previous studies reported that cyclinD1 could serve as a prognostic predictor in esophageal squamous cell carcinomas [13, 14]. A high expression of cyclinD1 is closely associated with cell proliferation of pancreatic cancer through analyzing human clinical tissues [15]. The survival of a cervical cancer patient is shorter when their cyclinD1 and survivin are overexpressed [16]. Elevated cyclinD1 can be downregulated by metastasis associated-1 knockdown to inhibit the cell proliferation and invasion of breast cancer [17]. CyclinD1 can function as a target of aspirin in resisting tamoxifen resistance in breast cancer [18]. BATF3/AP-1/cyclinD1signaling is a target of miR-760 in depressing the growth of colorectal cancer [19]. In glioblastoma, cyclinD1 can be targeted by miR-16 [20]. It remains unclear whether miR-520e could post-transcriptionally modulate cyclinD1 in breast cancer.

In our study, we tried to explore the function of tumor suppressive miR-520e in the modulation of cyclinD1 expression in breast cancer. Our finding shows that miR-520e is able to control the expression of cyclinD1 in breast cancer cells. For the regulatory mechanism investigation, we reveal that miR-520e can directly bind to the 3’UTR region of cyclinD1 to degrade its mRNA. In human clinical breast cancer tissues, we observed a negative correlation of miR-520e with cyclinD1. Our finding will emphasize the potential utilization of miR-520e and cyclinD1 in the treatment of breast cancer in the future.

## 2 Methods

### 2.1 Cell culture

Breast cancer cell line MCF-7 was grown in RPMI Medium 1640 (Gibco, USA) with 10% fetal bovine serum (Gibco) and maintained via a 5% CO2 incubator at 37°C.

### 2.2 MicroRNA mimic transfection

Lipofectamine 2000 (Invitrogen, USA) was used to transfect miR-520e and its antagonist, anti-miR-520e into MCF-7 cells. MiR-520e, anti-miR-520e and their control were bought from RiboBio (Guangzhou, China).

### 2.3 Patient tissues

Twenty cases of breast cancer tissues and paired noncancerous tissues were obtained from breast cancer patients with post-surgical written consents at the General Hospital of Tianjin Medical University (Tianjin, China) (Supporting Table S1). The Research Ethics Board has approved the study protocol used here.

### 2.4 RNA extraction, reverse transcription-polymerase chain reaction (RT-PCR) and quantitative real-time PCR

**Figure 1:**
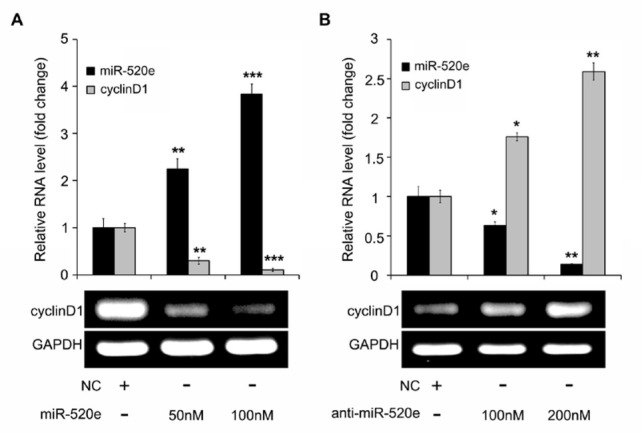
MiR-520e restrains the expression of cyclinD1 in breast cancer MCF-7 cells. (A, B) After miR-520e or anti-miR-520e was induced into MCF-7 cells, the level of cyclinD1 was determined by using RT-PCR assay of one-point detection in electrophoresis results and quantitative real-time PCR from a minimum of three different repeats for each sample. Meanwhile, the level of miR-520e in the cells transfected with miR-520e and anti-miR-520e was evaluated by quantitative real-time PCR. Statistical quantification from at least three independent experiments is included. *P <0.05; **P < 0.01; ***P < 0.001; Student’s *t* test.

**Figure 2:**
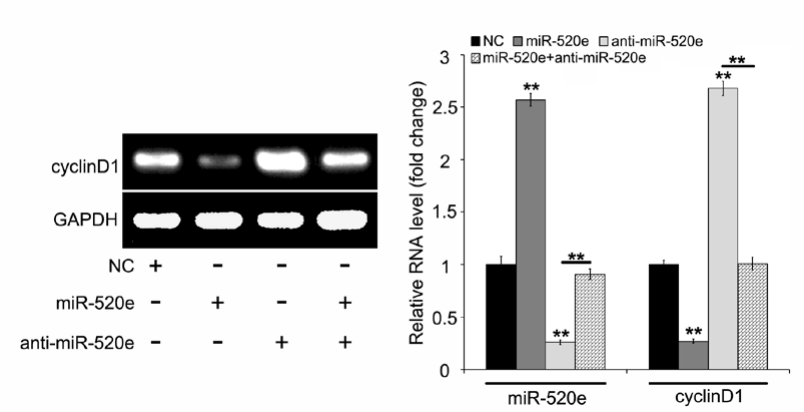
Anti-miR-520e reverses the inhibition of cyclinD1 induced by miR-520e. The expression of cyclinD1 at the mRNA level was tested by using RT-PCR assay of one-point detection in electrophoresis results and quantitative real-time PCR from a minimum of three different repeats for each sample. Meanwhile, the level of miR-520e in the cells transfected with miR-520e and anti-miR-520e was evaluated by quantitative real-time PCR when MCF-7 cells were treated with negative control mimics, miR-520e and/or anti-miR-520e. Statistical quantification from at least three independent experiments is included. *P <0.05; **P < 0.01; ***P < 0.001; Student’s t test.

The total RNA of breast cancer MCF-7 cells and clinical breast cancer samples was obtained by utilizing the TRIzol Reagent (Invitrogen, USA). For the detection of the cyclinD1 mRNA level, reverse transcription was performed by GoScript^TM^ Reverse Transcriptase (Promega, USA) and GAPDH was used as a loading control. Quantitative real-time PCR was performed using the QuantiNova SYBR Green PCR kit (Qiagen, Valencia, CA, USA). The relative cyclinD1 mRNA levels were analyzed by normalizing the threshold cycle (Ct) value to that of the internal loading control, GAPDH. For miR-520e, its level was measured using a TaqMan MicroRNA Reverse Transcription Kit (Thermo Fisher, Carlsbad, CA, USA) and TaqMan gene expression master mix (Thermo Fisher, Carlsbad, CA, USA) according to the manufacturer’s protocol. The level of miR-520e was analyzed by normalizing the threshold cycle (Ct) value to that of the internal loading control, U6 snRNA. In Figure 1 and 2, one-point detection of cyclinD1 was per

**Table 1:** Clinical characteristics of breast cancer samples

No.	Sex	Age	Pathology diagnosis
1	F	45	Breast cancer
2	F	38	Breast cancer
3	F	33	Breast cancer
4	F	47	Breast cancer
5	F	51	Breast cancer
6	F	39	Breast cancer
7	F	44	Breast cancer
8	F	30	Breast cancer
9	F	52	Breast cancer
10	F	59	Breast cancer
11	F	46	Breast cancer
12	F	48	Breast cancer
13	F	37	Breast cancer
14	F	52	Breast cancer
15	F	54	Breast cancer
16	F	49	Breast cancer
17	F	56	Breast cancer
18	F	55	Breast cancer
19	F	34	Breast cancer
20	F	43	Breast cancer

formed by RT-PCR assay and then run for electrophoresis. Also, the level of miR-520e and cyclinD1 was tested from a minimum of three different repeats for each sample through a quantitative real-time PCR assay after miR-520e or anti-miR-520e was transfected into breast cancer cells. In Figure 4, the level of miR-520e and cyclinD1 in clinical breast cancer tissues was tested from a minimum of three different repeats for each sample through a quantitative real-time PCR assay.

### 2.5 Constructs

pGL3-control vectors with a wild type or a mutant binding site between miR-520e and cyclinD1 3’UTR were constructed. The primers were used as follows: wt forward, 5’- GCTCTAGACCATTCCATTTCCAAGCACTTT-3’, reverse, 5’-GGGGGCCGGCC GGAAAGGAACTTATCATCCT-3’; mut forward, 5’- GCTCTAGAGGATAGCATTTCCTTCGT-GAAT-3’, reverse, 5’-GGGGGCCGGCC GGAAAGGAACTTAT-CATCCT-3’).

### 2.6 Luciferase reporter analysis

MCF-7 cells were seeded on 24-well plates. Luciferase reporter vectors (wt or mut) with microRNA mimics (or control) were transfected with Lipofectamine 2000 (Invitrogen). Firefly luciferase activities were normalized by Renilla luciferase activities. The Dual-Luciferase® Reporter Assay System (Promega, USA) was applied for testing luciferase activity.

### 2.7 Statistical analysis

A student’s t-test was used to measure the statistical significance between the two groups. A P value less than 0.05 was thought statistically significant. The data are presented as mean ± SD, and all experiments were performed independently a minimum of three times. The correlation between miR-520e and cyclinD1 expression in human clinical breast cancer tissues was evaluated by Pearson’s correlation coefficient.

## 3 Results

### 3.1 MiR-520e restrains the expression of cyclinD1 in breast cancer MCF-7 cells

At first, we were wondering whether tumor suppressor miR-520e affected oncogenic cyclinD1 expression in breast cancer MCF-7 cells. We transiently transfected miR-520e mimics obtained from RiboBio (Guangzhou, China) into MCF-7 cells. The data revealed that, compared with the negative control (NC) group, the level of cyclinD1 had obviously decreased under the treatment of the elevated dose of miR-520e in the cells. These results were obtained from a RT-PCR assay of one-point detection in electrophoresis results and quantitative real-time PCR from three different repeat at least for each sample (Figure. 1A). In addition, we found that the inhibitor of miR-520e, anti-miR-520e could induce the expression of cyclinD1 in the cells. These results were obtained from a RT-PCR assay of one-point detection in electrophoresis results and quantitative real-time PCR from three different repeat at least for each sample (Figure. 1B). Meanwhile, the level of miR-520e in the cells transfected with miR-520e and anti-miR-520e was evaluated by quantitative real-time PCR (Figure 1A and 1B). In future experiments, we want to evaluate the effect of anti-miR-520e on miR-520e and cyclinD1 in breast cancer cells. Our results showed that miR-520e inhibited the level of cyclinD1 and then anti-miR-520e enhanced the expression of cyclinD1. Furthermore, the reintroduction of miR-520e in anti-miR-520e-treated cells could destroy the augmentation of cyclinD1 by anti-miR-520e in MCF-7 cells by using an RT-PCR assay of one-point detection in electrophoresis results and quantitative real-time PCR from a minimum of three different repeats for each sample (Figure. 2). At the same time, the overexpression or inhibition of miR-520e in the cells was confirmed by quantitative real-time PCR (Figure 2). Our finding implies that the tumor suppressor miR-520e can control the expression of cyclinD1 in breast cancer cells.

### 3.2 MiR-520e binds to the 3'UTR of cyclinD1 mRNA to reduce its level

**Figure 3:**
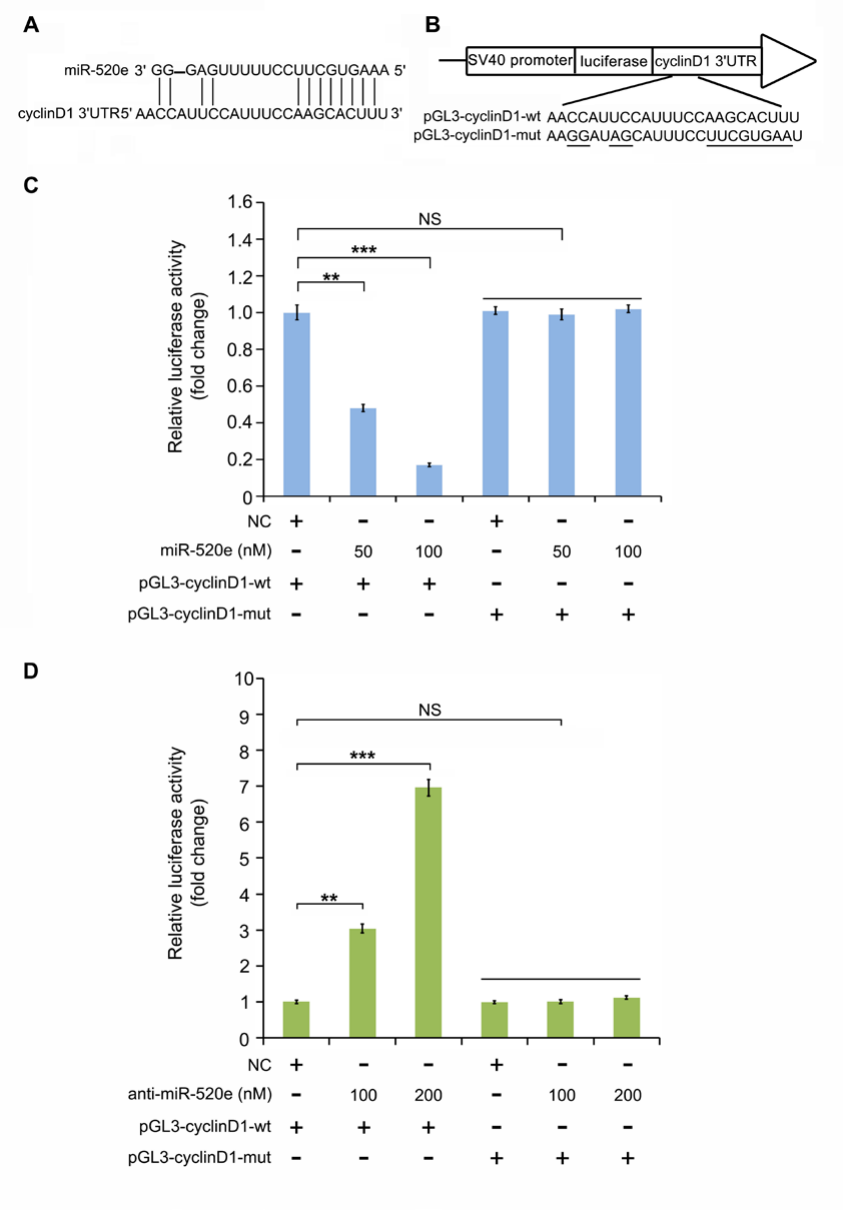
MiR-520e binds to the 3'UTR of cyclinD1 mRNA to reduce its level. (a) (A, B) The binding between miR-520e and the 3¢UTR of cyclinD1 mRNA is presented. The luciferase vector containing wild type and mutant binding sites within the 3¢UTR of cyclinD1 (wt or mut) with miR-520e is constructed. (C) The regulation of miR-520e on the luciferase activity of pGL3-cyclinD1-wt or pGL3-cyclinD1-mut was revealed through luciferase reporter assay in breast cancer MCF-7 cells. (D) The regulation of anti-miR-520e on the luciferase activity of pGL3-cyclinD1-wt or pGL3-cyclinD1-mut was revealed through luciferase reporter assay in breast cancer MCF-7 cells. NS, not significant; **P < 0.01; ***P < 0.001; Student’s t test.

To investigate how miR-520e regulates cyclinD1 in breast cancer cells, we searched for the binding sites between miR-520e and cyclinD1 through online software (http://www.targetscan.org) The binding site of miR-520e exists within the 3’ untranslated region (3’UTR) of cyclinD1 mRNA (Figure 3A). We next constructed the luciferase reporter vectors containing a wild-type or a mutant binding site of miR-520e with the 3’UTR of cyclinD1 mRNA (pGL3-cyclinD1-wt or pGL3-cyclinD1-mut) (Figure. 3B). Taking a step further, we examined whether miR-520e could weaken the luciferase activities of pGL3-cyclinD1-wt or pGL3-cyclinD1-mut. We observed that under treatment with an increased concentration of miR-520e, the luciferase activities of pGL3-cyclinD1-wt sharply decreased. When pGL3-cyclinD1-mut was introduced into the cells, miR-520e failed to affect the luciferase activities (Figure. 3C). However, anti-miR-520e could induce the luciferase

**Figure 4:**
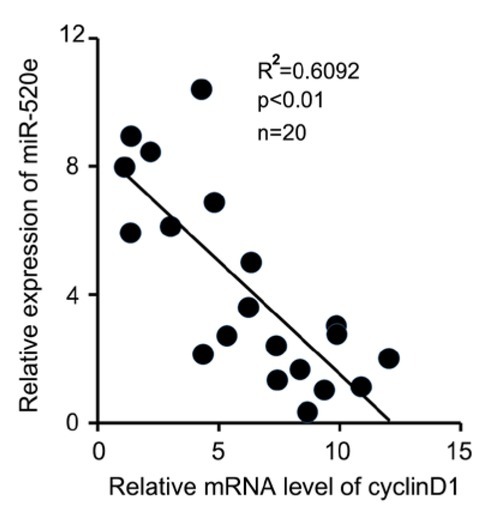
In human clinical breast cancer tissues, miR-520e is positively correlated with cyclinD1. The correlation of miR-520e and cyclinD1 was analyzed in clinical breast cancer tissues (Pearson′s correlation coefficient, R2=0.6092).

activities of pGL3-cyclinD1-wt. But it lost the control of pGL3-cyclinD1-mut (Figure. 3D). So, all our data indicate that cyclinD1 is a novel target gene of miR-520e in breast cancer.

### 3.3 In human clinical breast cancer tissues, miR-520e positively correlates with cyclinD1

CyclinD1 is overexpressed and implicated in the development of many types of cancers [14, 15, 16, 17, 18]. Researchers have presented evidence that miR-520e can play an inhibitory role during the progression of some cancers [1, 4, 5, 9, 10]. We want to know the correlation between miR-520e and its target gene, cyclinD1 in clinical breast cancer tissues. Twenty cases of human clinical breast cancer samples were utilized. Through a quantitative real time-PCR assay from a minimum of three different repeats for each sample, we found that in cyclinD1-upregulated breast cancer tissues the level of miR-520e was decreased and then in cyclinD1-downregulated breast cancer tissues miR-520e was elevated. Moreover, our data demonstrated that there was a negative correlation between the expression of cyclinD1 and the level of miR-520e in clinical breast cancer samples (Pearson′ correlation coefficient R^2^=0.6092, p < 0.01) (Figure. 4). Altogether, our finding presents that in clinical breast cancer samples, low miR-339 correlates with high cyclinD1.

## 4 Discussion

Among women, breast cancer always remains the most common cause of cancer mortality all around the world [21]. As one type of small non-coding RNA, microRNAs can directly interact with their target genes to promote the degradation of mRNA or the inhibition of translation, which may serve as a cancer promoter or cancer suppressor in different types of cancers including breast cancer. MiR-520e can suppress breast cancer through making breast cancer cells sensitive to complement attacks [1]. Some microarray data shows the correlation of miR-520e with the occurrence and development of breast cancer[2]. As an important cell cycle driver, cyclinD1 encoded by CCND1 is a prognostic and predictive factor in cancers. Elevated cyclinD1 can be downregulated by metastasis associated-1 knockdown to inhibit the cell proliferation and invasion of breast cancer [17]. CyclinD1 can function as a target of aspirin in resisting tamoxifen resistance in breast cancer [18]. However, the role of miR-520e in the modulation of cyclinD1 still remains unclear.

In our investigation, we firstly wanted to know whether tumor suppressive miR-520e can function in the regulation of oncogenic cyclinD1 in breast cancer. We synthetized miR-520e mimics and then transduced them into breast cancer cells. We observed that an elevated concentration of miR-520e could dramatically decrease the level of cyclinD1 in breast cancer MCF-7 cells. Meanwhile, the antagonist of miR-520e, anti-miR-520e induced the expression of cyclinD1. To further confirm the regulation of miR-520e on the expression of cyclinD1, we introduced miR-520e into the cells after its antagonist was transfected. As expected, miR-520e weakened the upregulation of cyclinD1 mediated by anti-miR-520e in the cells through quantitative real time-PCR analysis. To clarify how miR-520e modulates the expression of cyclinD1 at the post-transcription level, we used online software to predict the binding sites between miR-520e and cyclinD1 mRNA. The binding site of miR-520e is within the 3’UTR of cyclinD1 mRNA and we cloned the luciferase reporter vectors containing wild-type or mutant binding sites between miR-520e and cyclinD1 mRNA 3’UTR. We observed that miR-520e or anti-miR-520e obviously inhibited or induced the luciferase activities of a wild-type reporter vector and lost control over the mutant reporter vector. In human clinical breast cancer tissues, we analyzed the correlation of miR-520e and cyclinD1. The data showed a negative correlation between the level of miR-520e and the expression of cyclinD1 in breast cancer tissues. So, our finding proves that miR-520e can regulate the expression of cyclinD1 at the level of post-transcription in breast cancer.

In conclusion, we firstly report that oncogenic cyclinD1 is a novel target gene of tumor suppressor miR-520e in breast cancer. MiR-520e is capable of directly binding to the 3’UTR of cyclinD1 mRNA to promote the degradation of cyclinD1 mRNA, leading to the inhibition of cyclinD1 in breast cancer. Therapeutically, our finding provides new evidence and support for the utilization of miR-520e and cyclinD1 in breast cancer treatment.
